# Nanolignin by Ultrasonication: Tuning the Process for Tailored Materials Characteristics

**DOI:** 10.1021/acsomega.5c03665

**Published:** 2025-06-21

**Authors:** Eleonora Ruffini, Emanuela Bellinetto, Stefano Turri, Gianmarco Griffini

**Affiliations:** Department of Chemistry, Materials and Chemical Engineering “Giulio Natta”, 18981 Politecnico di Milano, Piazza Leonardo da Vinci 32, Milano 20133, Italy

## Abstract

In this work, a Design-of-Experiments (DoE) methodology was adopted to study and optimize the ultrasonication process of lignin in water to obtain lignin nanoparticles (LNPs) so as to establish qualitative and quantitative cause–effect relationships between the operating variables (i.e., lignin concentration, processing time, and sonication amplitude) and the distinctive features of the so-obtained nanostructured materials. Monitoring the evolution of the chemical, physical, morphological, and thermal characteristics of lignin upon ultrasonic irradiation enabled the identification of the most relevant process parameters (viz., factors) affecting the average particle size and shape, concentration of hydroxyl/carboxyl groups, and suspended fraction of particles, which were taken as the target materials characteristics (viz., systems responses). Interestingly, the selection of suitable process conditions allowed to obtain exclusively spherical LNPs with perfectly circular shape profile, thus providing the first demonstration of the formation of lignin nanospheres through a size-reduction method not relying on the use of solvents or other chemicals. Furthermore, highly predictive fitting models were generated and validated to accurately and reliably estimate the system responses at any experimental point within the investigation range. As a validation of such process optimization, poly­(vinyl alcohol) (PVA) films incorporating up to 20% (w/w) of either pristine or ultrasound-treated lignin were prepared so as to evaluate the effect of tailored lignin (nano)­particle characteristics on the chemical, morphological, thermal, and mechanical properties of the resulting (nano)­composite systems. The nanometric size of LNPs, together with their spherical morphology and lower molecular weight vs pristine systems was found to foster stronger matrix–filler interactions and improved distributive mixing within the PVA matrix, ultimately leading to higher-performance nanocomposite materials. This work provides new insights into the production of LNPs via ultrasonication, demonstrating that precise control over their size, morphology, and chemical functionalities can be predictively attained by the optimal tuning of relevant process parameters.

## Graphical abstract

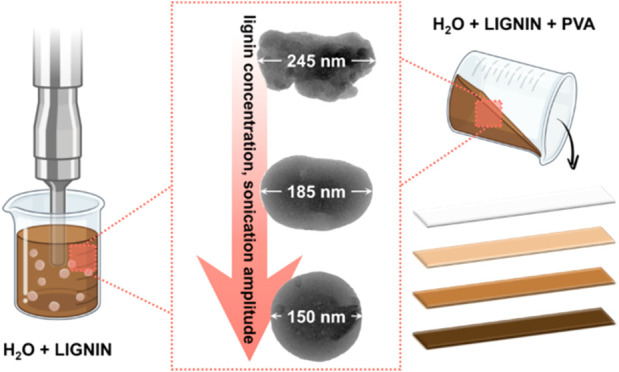

## 1 Introduction

With its structure formed by the radical coupling of three phenylpropanoid units (i.e., *p*-hydroxyphenyl (H), guaiacyl (G), and syringyl (S)), lignin is the most abundant natural aromatic biopolymer on Earth, constituting 15–30% of the dry mass of plants, depending on the species.^1–3^ It is estimated that more than 50 million tons per year of lignin are generated as a byproduct of the pulp and paper industry, 95% of which is incinerated as a low-value waste for energy recovery.^4^ These data spotlight the need for developing and implementing strategies for turning such an unexploited, yet full of potential, biomaterial into high-value-added products.

Being rich in hydroxyl (both aliphatic and aromatic ones), carboxyl, and carbonyl groups, which may promote favorable covalent and noncovalent interactions with other polymers, lignin lends itself well to being used as a reinforcing filler. In this regard, lignin-based composites have been described in several research works in the past decade, highlighting that preprocessing lignin prior to its introduction into the polymeric matrix is a pivotal step to enhance the interaction between the two phases and, ultimately, the physical properties of the final material.^5,6^

An effective incorporation of lignin particles into a polymeric carrier to obtain lignin-based composite systems can be achieved either upon chemical modification of lignin to introduce new functional groups that improve matrix−filler interactions or upon size reduction of lignin to increase the specific surface area in contact with the continuous (polymeric) phase. This latter compatibilization strategy can be implemented following either chemical or physical routes, both aimed at obtaining a nanosized reinforcing filler capable of effective interfacial adhesion and load transfer to the host matrix.^7^

Chemical (or bottom-up) methods typically achieve the self-assembly of solubilized lignin macromolecules into spherical nanoparticles by leveraging lignin amphiphilic nature, that is, its ability to organize itself into energy-minimizing structures through hydrophobic (i.e., π–π stacking and van der Waals forces) and hydrophilic (i.e., hydrogen bonding) interactions.^2,8^ This mechanism can be triggered by processes such as antisolvent precipitation and solvent exchange/solvent shifting, which allow for good control over particle morphology and size distribution. However, both of them often rely on the need for organic chemicals and multiple steps, ultimately leading to toxicological concerns, nonnegligible operational costs, and low yields.^2,9,10^

Physical (or top-down) approaches involve the fragmentation and breaking down of larger lignin agglomerates into smaller isolated particles. These techniques rely solely on the action of mechanical forces (primarily shear forces, generated by processes such as ball milling and high-shear homogenization, or cavitation by ultrasonication) that promote collisions among particles, as well as surface erosion and volume pulverization phenomena.^5,11,12^ Compared to their chemical counterpart, these methods present some advantages associated with their more favorable toxicological profile (lignin is either used as is or dispersed in water, typically with no use of critical solvents), straightforward process implementation (little to no downstream processing is required), and higher processable volumes (a prerequisite for industrial scale-up).^2,3,5,11,12^ However, the obtained nanoparticles are usually characterized by the irregular shape and broad size distribution.^2,3,5,11,12^

Among the aforementioned top-down approaches, ultrasonication is a technique that has recently garnered growing interest within the scientific community. Several research works on ultrasound-assisted preparation of LNPs and their incorporation into polymer-based nanocomposite systems have been reported in the literature, with only a few of them conducting process studies aimed at optimizing key output variables of interest (typically hydrodynamic diameter and zeta potential) for predictive control over LNP characteristics.^3,5,11,13^ In particular, when dealing with the ultrasonic irradiation of aqueous suspensions of commercial lignin, in the absence of any chemical agents and/or solvents, a thorough understanding of how process conditions can influence the chemical–physical properties of the resulting nanoparticles (such as regular/irregular shape and hydroxyl/carboxyl group content) is still lacking, despite their strategic importance in view of lignin exploitation as a macromolecular building block for value-added biobased materials and products. An in-depth analysis of this topic requires the adoption of a statistical methodology, enabling one to identify key correlations between process parameters and LNP characteristics, as well as to attain a predictively controlled modification of lignin particles, not only in terms of size and colloidal stability, but also in terms of morphology, chemical functionality, and physical response, by manipulating easily accessible operating variables.

To bridge this gap, a Design-of-Experiments (DoE) methodology was set up and implemented in this work to investigate the ultrasonic treatment of softwood lignin from kraft pulping, taken as an industrially relevant representative platform of such polyphenolic biobased macromolecules.^14,15^ The analysis was based on a Box–Behnken design (BBD) with three factors, three levels, and three replicates of the central point, where the lignin concentration in water (1–5 wt %), processing time (4–12 h), and sonication amplitude (50–90%) were selected as most relevant input variables. This approach provided qualitative and quantitative insights into their mutual interactions as well as into their effects on key output variables of interest, namely, the average particle size, chemical functionality, average particle shape circularity, and mass fraction of suspended particles in water after the ultrasonic process. The chemical, physical, and thermal properties of ultrasound-treated lignin-in-water suspensions and LNPs were quantified and compared with those featured by the untreated parent material. Based on the outcomes of this process optimization, the exploitability of the so-obtained nanoparticles as biofiller in polymer matrices was explored. Given its excellent biodegradability, water solubility, film-forming capability, and compatibility with lignin, poly­(vinyl alcohol) (PVA) was identified as one of the most promising candidates for this purpose, also in view of its extremely versatile applicability in several industrial sectors (e.g., packaging, electronics, cosmetics, agricultural applications, *etc.*).^16^ Therefore, PVA-based films incorporating 5% (w/w), 10% (w/w), and 20% (w/w) of either pristine or suitably ultrasound-treated lignin were prepared to evaluate the effect of tailored lignin (nano)­particle characteristics on the chemical, morphological, thermal, and mechanical properties of the resulting (nano)­composite systems.

## 2 Materials and Methods

### 2.1 Materials

Indulin AT, a commercial kraft pine lignin, was supplied by Ingevity (North Charleston, SC). Poly­(vinyl alcohol) (PVA) was supplied by MonoSol (Kuraray Plastics Co., Ltd., Chiyoda, Tokyo, Japan). All other reagents and solvents were purchased from Merck (Merck KGaA, Darmstadt, Germany) and used without any further purification, if not otherwise specified.

### 2.2 Design of Experiments (DoE)

A DoE approach was adopted to systematically investigate the potential effects of process parameters on chemical and physical properties of LNPs. Lignin concentration (*C*) in water varying in the 1–5 wt % range, processing time (*t*) between 4 and 12 h, and sonication amplitude (*A*) varying between 50 and 90% were selected as independent variables (factors). The combinations of factors were established based on a BBD, which requires a number of experiments (*N*) defined by eq 1

1 $$N=k^{2}+k+c_{p}$$

where *k* is the number of factors, and *c*_*p*_ is the number of replicates of the central point.^17,18^

In the present work, three levels and three replicates of the central point were considered, leading to a total of 15 runs (Table 1).

**Table 1:** The BBD with Three Factors and Three Levels Planned and Conducted in This Work

standard order	*C* (wt %)	*t* (h)	*A* (%)	sample^a^
1	1 (−1)	4 (−1)	70 (0)	1–4–70
2	5 (+1)	4 (−1)	70 (0)	5–4–70
3	1 (−1)	12 (+1)	70 (0)	1–12–70
4	5 (+1)	12 (+1)	70 (0)	5–12–70
5	1 (−1)	8 (0)	50 (−1)	1–8–50
6	5 (+1)	8 (0)	50 (−1)	5–8–50
7	1 (−1)	8 (0)	90 (+1)	1–8–90
8	5 (+1)	8 (0)	90 (+1)	5–8–90
9	3 (0)	4 (−1)	50 (−1)	3–4–50
10	3 (0)	12 (+1)	50 (−1)	3–12–50
11	3 (0)	4 (−1)	90 (+1)	3–4–90
12	3 (0)	12 (+1)	90 (+1)	3–12–90
13	3 (0)	8 (0)	70 (0)	3–8–70 (1)
14	3 (0)	8 (0)	70 (0)	3–8–70 (2)
15	3 (0)	8 (0)	70 (0)	3–8–70 (3)

a The samples are labeled as *X*–*Y*–*Z*, where “*X*” indicates the lignin concentration (i.e., *C* (wt %)), “*Y*” indicates the processing time (i.e., *t* (*h*)), and “*Z*” indicates the sonication amplitude (i.e., *A* (%)).

This experimental design enables the estimation of the system response at any experimental point within the investigation range.^18,19^ The predicted response can be calculated using the response function that can be represented as a regression equation in the following form

2 $$y=\beta_{0}+k\sum_{i=1}^{k}\beta_{i}x_{i}+\sum_{i=1}^{k}\beta_{\mathrm{ii}}x_{i}^{2}+\sum_{j}\sum_{<i=2}^{k}\beta_{\mathrm{ij}}x_{i}x_{j}$$

where *y* is the response; *x*_*i*_ and *x*_*j*_ are variables (*i* and *j* range from 1 to *k*); β_0_ is the model intercept of coefficient; β_*i*_, β_*ii*_, and β_*ij*_ are the interaction coefficients of linear, quadratic, and second-order terms, respectively; and *k* is the number of independent parameters (in the present work, *k* = 3).^18,20^

Minitab software (version 21.4.2.0, Minitab, Philadelphia, PA) was used to assess potential effects of the ultrasound treatment on lignin. To this end, the following parameters were selected as dependent variables (responses): (1) average particle size (*d*_average_); (2) particle-size polydispersity index (PDI_DLS_); (3) glass-transition temperature (*T*_g_); (4) hydroxyl and carboxyl functionalities (OH and COOH, respectively); (5) average particle shape circularity (Π); and (6) mass fraction of suspended lignin particles in water after the ultrasonic process (*x*_lig,sus_). It is worth noting that PDI_DLS_ and *T*_g_ were not included in the set of responses for the statistical analysis, as will be discussed in detail in Section 3.1.

### 2.3 Lignin Nanoparticles Production

Ultrasonication was performed by means of a VCX 500 ultrasonic processor equipped with a 13 mm diameter probe (Sonics & Materials, Inc., Newton, CT). All of the samples were prepared by suspending a proper amount of lignin in 50 mL of distilled water, with the aid of magnetic stirring to favor the dispersion of the powder in the liquid. Prior to the treatment, lignin was dried under vacuum overnight at 40 °C to remove any traces of moisture. Ultrasound-treated suspensions were characterized and then freeze-dried to recover the dry nanoparticles, which were analyzed as well to assess potential effects of ultrasound treatment on raw kraft lignin.

### 2.4 Lignin Nanoparticles Characterization

Differential scanning calorimetry (DSC) was used to determine the *T*_g_ of both raw kraft lignin and freeze-dried nanoparticles. DSC curves were collected on ∼15 mg samples by means of a DSC823e instrument (Mettler-Toledo, Columbus, OH). The analyses were performed under a nitrogen flux at a scan rate of 20 °C/min using the following three-step run: (i) heating from 25 to 150 °C; (ii) cooling from 150 to 25 °C; and (iii) heating from 25 to 250 °C. The value of *T*_g_ was determined as the inflection point of the DSC trace recorded in the second heating run.

Dynamic light scattering (DLS) was used to determine the average particle size (i.e., the intensity-weighted mean hydrodynamic size) and particle-size polydispersity index. DLS measurements were performed by means of a Zetasizer Nano analyzer (Malvern Panalytical Ltd., Malvern, UK) at 25 °C. All of the samples were prepared by diluting ultrasound-treated nanolignin suspensions until reaching a laser obscuration of 30–40%. In the field of nanoparticular characterization, the polydispersity index is a dimensionless measure of the broadness of the size distribution of a sample.^21^ As established by International standards organizations (ISOs), PDI_DLS_ values can vary from 0–0.07 for monodispersed samples to 0.7–1 in the case of broad particle-size distributions.^21^

Phosphorus-31 nuclear magnetic resonance (^31^P NMR) spectroscopy was used to quantify the amounts of hydroxyl and carboxyl functionalities in both raw kraft lignin and freeze-dried ultrasound-treated nanoparticles. ^31^P NMR spectra were collected by means of a Bruker Avance III HD 400 MHz spectrometer (Bruker, Billerica, MA). The analyses were performed at 27 °C, 1 s acquisition time, a 5 s relaxation delay, and 256 scans. All of the samples were prepared according to a procedure described in the literature.^22^ Mnova software (version 6.0.2, Mestrelab Research, S.L., Santiago de Compostela, Spain) was used for the acquisition and data treatment. The signals in the regions 150–147, 145–137, and 137–135 ppm were integrated and assigned to the aliphatic hydroxyl, aromatic hydroxyl, and carboxyl groups, respectively.

Average particle shape circularity was considered as a two-dimensional indicator of the sphericity of the nanoparticles produced through ultrasounds, and it was calculated according to eq 3

3 $$\Pi =4\times \pi \times \frac{A}{p^{2}}$$

where *A* and *p* are the area and perimeter of the particle, respectively.^23^ More regular/circular particle profiles are associated with higher Π values, until reaching a maximum value of 1, attributed to a perfectly circular shape.^23^ A drop of suspension was deposited on a lacey carbon-supported copper grid and left to dry at room temperature. Micrographs of lignin (nano)­particles were acquired by means of a CM200 FEG transmission electron microscope (TEM) (Philips N.V., Amsterdam, Netherlands) equipped with a field emission gun operated at 200 keV accelerating voltage and were digitally analyzed by ImageJ software (version 1.8.0, National Institutes of Health, Bethesda, MD). For each sample, Π was calculated as the average value out of at least 30 measurements (i.e., 30 different nanoparticles).

The fraction of suspended lignin particles was defined as the ratio between lignin concentration (wt %) in the dispersing medium before and after ultrasonication, respectively, and it was calculated according to eq 4

4 $$x_{\mathrm{lig},\mathrm{sus}}=\frac{C_{\mathrm{actual}}}{C_{\mathrm{nominal}}}$$

where *C*_actual_ is the lignin concentration in the ultrasound-treated suspension, and *C*_nominal_ is the initial lignin concentration in the dispersing medium (the concentration was evaluated as the mass ratio between lignin and distilled water).

After a 72-h resting period following the end of the ultrasonic treatment, a known volume of suspension was withdrawn and left to dry under heating (40 °C in a vacuum oven). The sample was weighed several times until reaching a plateau value, indicating that water was completely evaporated. *C*_actual_ was determined as the mass ratio between the amount of lignin in the sample and the amount of water in the withdrawn volume of the ultrasound-treated suspension. For each suspension, *x*_lig,sus_ was calculated as the average value of at least three measurements.

Gel permeation chromatography (GPC) was used to estimate the number- and weight-average molecular weights (*M_n_* and *M*_w_, respectively) of the freeze-dried, ultrasound-treated lignin nanoparticles. GPC chromatograms were collected on 200 μL samples (concentration: 2 mg/mL) by means of a 510 HPLC system (Waters Corporation, Milford, Massachusetts, MA) equipped with a 515 HPLC pump (Waters Corporation, Milford, Massachusetts, MA), a 2410 refractive index detector (Waters Corporation, Milford, Massachusetts, MA), and four Styragel columns (models: HR 5, HR 4, HR 3, and HR 2; dimensions: 7.8 mm (inner diameter) × 300 mm (length)) (Waters Corporation, Milford, Massachusetts, MA) connected in series and packed with 5 μm spherical particles. The analyses were performed at 35 °C at a flow rate of 1 mL/min using tetrahydrofuran (THF) as an eluent. A calibration curve was constructed using monodispersed fractions of polystyrene (molecular weight: 10^2^–10^4^ g/mol). Prior to analysis, all of the samples were acetylated by following a procedure described in previous works.^24,25^

### 2.5 PVA-Based Films Preparation

Aqueous solutions were prepared by solubilizing PVA in distilled water at 15 wt % at room temperature under magnetic stirring to allow for complete homogenization of the system. Neat PVA films were obtained upon casting, solvent evaporation under natural convection, and vacuum drying at 60 °C until reaching a constant weight. PVA/lignin composite films were prepared by directly adding proper amounts of raw kraft lignin to the aqueous PVA solutions, while a slightly different protocol was followed to obtain systems incorporating LNPs. In this latter case, with the aim of ensuring the correct filler loading within the final materials while maintaining a PVA concentration of 15 wt % with respect to water, ultrasound-treated nanolignin aqueous suspensions were prepared, properly diluted, and directly used to solubilize PVA. The obtained films were labeled as PVA, PVA/KLxx, and PVA/LNPxx, where “KL” and “LNP” stand for raw kraft lignin and ultrasound-treated LNPs, respectively, while “xx” indicates the filler loading expressed as weight percentage with respect to PVA.

### 2.6 PVA-Based Films Characterization

Attenuated total reflection-Fourier transform infrared (ATR-FTIR) spectroscopy was used to study the intermolecular interactions between PVA and lignin or nanolignin in the produced composite and nanocomposite films. IR spectra were collected by means of a Nicolet iS50 FTIR spectrometer (Thermo Fisher Scientific Inc., Waltham, MA) equipped with an ATR accessory. These analyses were performed at room temperature in air by recording 64 scans at a resolution of 4 cm^–1^ in the wavenumber range of 4000–600 cm^–1^. All spectra were normalized with respect to the signal at 1515 cm^–1^ related to lignin aromatic ring vibrations and taken as an invariant band.

Scanning electron microscopy (SEM) was used to examine the morphology of the PVA-based films with a focus on the distribution of the biobased filler in the polymer matrix. All specimens were cryofractured with liquid nitrogen, and the so-obtained cross sections were metalized with gold prior to analysis. SEM images were acquired by means of an EVO 50 scanning electron microscope (Carl Zeiss AG, Oberkochen, Germany) operated at 15 kV.

Differential scanning calorimetry (DSC) was used to determine the *T*_g_, melting temperature (*T*_m_), melting enthalpy (Δ*H*_m_), and crystallinity degree (*χ*_c_) of the PVA-based films. DSC curves were collected on ∼15 mg samples by means of a DSC823e instrument (Mettler-Toledo, Columbus, OH). The analyses were performed under a nitrogen flux at a scan rate of 20 °C/min, using a three-step run including (i) heating from 25 to 125 °C; (ii) cooling from 125 to 25 °C; and (iii) heating from 25 to 250 °C. *T*_g_ and *T*_m_ were determined as the inflection point and the peak temperature, respectively, of the DSC trace recorded in the second heating run. *χ*_c_ was calculated according to eq 5

5 $$\chi_{c}=\frac{\Delta H_{m}}{\Delta H_{m}^{0}\times \left(1-\varphi \right)}\times 100$$

where Δ*H*_m_ is the measured melting enthalpy of the sample; Δ*H*_m_^0^ is the melting enthalpy of 100% crystalline PVA (i.e., 161.6 J/g), and φ is the mass fraction of lignin in the sample.^26^

Thermogravimetric analysis (TGA) was used to study the thermooxidative stability of the PVA-based films. TGA curves were collected on ∼20 mg samples by means of a TGA 550 instrument (TA Instruments, New Castle, DE). The analyses were performed in air at a scan rate of 20 °C/min from 25 to 800 °C.

Uniaxial tension testing was used to determine the tensile properties of the PVA-based films in compliance with the ISO 527-3 standard. Stress–strain curves were collected on 15 × 150 cm^2^ strip-shaped specimens by means of a Z010 universal testing machine (Zwick GmbH & Co., Ulm, Germany) equipped with a 10 kN load cell and two extensometers. Tests were performed at room temperature, at a crosshead speed of 10 mm/min, and with an initial distance between the grips of 100 mm. Data were recorded by means of testXpert software (version 3.4.0.4452, Zwick GmbH & Co., Ulm, Germany). For each formulation, a minimum of five specimens were tested.

## 3 Results and Discussion

### 3.1 Box–Behnken Design on the Lignin Ultrasonication Process

With the aim of investigating statistically significant correlations between lignin ultrasonication-process parameters (viz., factors) (namely, lignin concentration (*C*) in the dispersing medium, processing time (*t*), and sonication amplitude (*A*)) and chemical–physical properties of the resulting nanoparticles (viz., responses) (namely, average particle size (*d*_average_), particle-size polydispersity index (PDI_DLS_), glass-transition temperature (*T*_g_), hydroxyl and carboxyl functionalities (OH and COOH, respectively), average particle shape circularity (Π), and mass fraction of suspended lignin particles in water after the ultrasonic process (*x*_lig,sus_)), a Design-of-Experiments (DoE) methodology was adopted. Corresponding levels for each factor were selected with the final purpose of defining a previously unexplored experimental domain. In particular, lignin concentration (*C*) was varied between 1 and 5 wt %, since several works have assessed the effects of ultrasonic irradiation on aqueous suspensions at lignin concentrations up to 1 wt %, while no other studies have extended the investigation domain up to values included within the interval considered in the present DoE. The same rationale holds for the processing time (*t*) ranging between 4 and 12 h, with shorter treatments being widely reported in the literature, while longer ones being worthless in view of a potential industrial exploitation. Finally, the sonication amplitude (*A*) value span (i.e., 50–90%) was chosen with the objective of evaluating how LNP final properties changed upon medium-to-high intensity ultrasonic irradiation. The experiments were planned, conducted, and analyzed based on a Box–Behnken design (BBD), deemed as the best-suited option to the scope of our investigation, for its efficiency in identifying cause-and-effect relationships between factors and responses, as well as second-order interaction effects among factors while requiring fewer runs and milder operating conditions compared to other designs.^27–30^

After a preliminary analysis of data to check whether all of the output variables were dependent on the input variables, the particle-size polydispersity and the glass-transition temperature of the obtained LNPs after the ultrasonic treatment were not found to vary significantly among the samples (Table S3). In particular, values of PDI_DLS_ ranging from 0.188 to 0.393 were recorded on all of the treated suspensions, which is indicative of acceptably homogeneous and moderate particle-size distributions regardless of the lignin concentration, processing time, and sonication amplitude. Similarly, all of the analyzed ultrasonication-process conditions yielded LNP samples showing *T*_g_ values in the range of 157–163 °C, in close proximity to that of the untreated parent material (160 °C). Accordingly, it was concluded that the ultrasonic treatment had no significant effect on the lignin transition from the glassy to the rubbery state, which occurred roughly in the same temperature range before and after ultrasonication.

For all other system responses, a fitted model was computed using Minitab software (regression coefficients and the coefficient of determination (*R* ^2^) for each response function are reported in Table S2). All regression equations generated as output of the statistical analysis exhibited *R* ^2^ values > 0.91, indicating good fit and accuracy of the defined models. The fitted models were also empirically validated by checking the reliability of the predicted values. To this end, two additional lignin ultrasonication experiments falling within the investigation range were performed and the observed responses were compared to the estimated ones. These validation tests were conducted using the following combinations of factors and levels (viz., process conditions): *C* = 2 wt %, *t* = 6 h, *A* = 60% (condition 2–6–60); *C* = 4 wt %, *t* = 10 h, *A* = 80% (condition 4–10–80). As shown in Table 2, an excellent match is observed between the actual and the predicted average values for the target responses in both validation experiments, confirming that the fitted models are accurate and reliable, and that they can be used as predictive tools for the estimation of the system response at any experimental point within the investigation range.

**Table 2:** Actual and Predicted System Responses for the 2–6–60 and 4–10–80 Experiments

		sample	
response	value^a^	2–6–60	4–10–80
*d*_average_ (nm)	actual	207 ± 10	161 ± 8
	predicted	212 ± 15	158 ± 15
OH (mmol/g)	actual	5.53 ± 0.03	5.47 ± 0.03
	predicted	5.53 ± 0.03	5.47 ± 0.03
COOH (mmol/g)	actual	0.32 ± 0.02	0.32 ± 0.02
	predicted	0.35 ± 0.02	0.34 ± 0.02
Π (−)	actual	0.75 ± 0.05	0.95 ± 0.01
	predicted	0.76 ± 0.17	0.96 ± 0.17
*x*_lig,sus_ (−)	actual	0.70 ± 0.03	0.85 ± 0.03
	predicted	0.68 ± 0.05	0.87 ± 0.05

a The actual responses are presented as average values ± standard deviation out of at least three measurements. The predicted responses are reported as fitted values ± their respective prediction interval based on the estimation computed by Minitab software.

Based on this preliminary investigation evidencing the most relevant factors and highlighting the statistical soundness of the proposed model, a detailed cause–effect analysis was carried out focusing on the role of lignin concentration in the water dispersing medium, processing time, and sonication amplitude on the definition of the output variables of interest, namely, average particle size, chemical functionality, average particle shape circularity, and fraction of suspended particles.

DLS measurements were performed to determine the intensity-weighted mean hydrodynamic size (i.e., *Z*-average size) for each ultrasound-treated aqueous LNPs suspension. Particle-size reduction in a colloidal system subjected to strong ultrasonic irradiation is caused by two main phenomena: on the one hand, volume pulverization produced by cavitation microjets and shockwaves, which breaks larger agglomerates into smaller particles; on the other hand, interparticle collisions and surface erosion caused by microstreaming and shear forces, which abrade particle edges and further decrease their dimension.^31,32^

In the present work, regardless of the duration and intensity of the ultrasonication process, experiments at the same level of lignin concentration resulted in roughly the same *d*_average_ value. This evidence suggests that the nominal concentration of lignin in the water dispersing medium plays a key role in determining the average dimension of the nanoparticles recovered after the treatment. These considerations are confirmed by the results of the DoE analysis, as illustrated by the Pareto chart of the standardized effects and the main effects plot for *d*_average_ shown in Figure 1. In particular, the red dotted line on the Pareto chart (Figure 1a) indicates which effects are statistically significant at the 0.05 level with the current model terms, and it is drawn at a value corresponding to the (1 – α/2) quantile of a t-distribution with degrees of freedom equal to the degrees of freedom for the error term (i.e., 5).^33^

**Figure 1:**
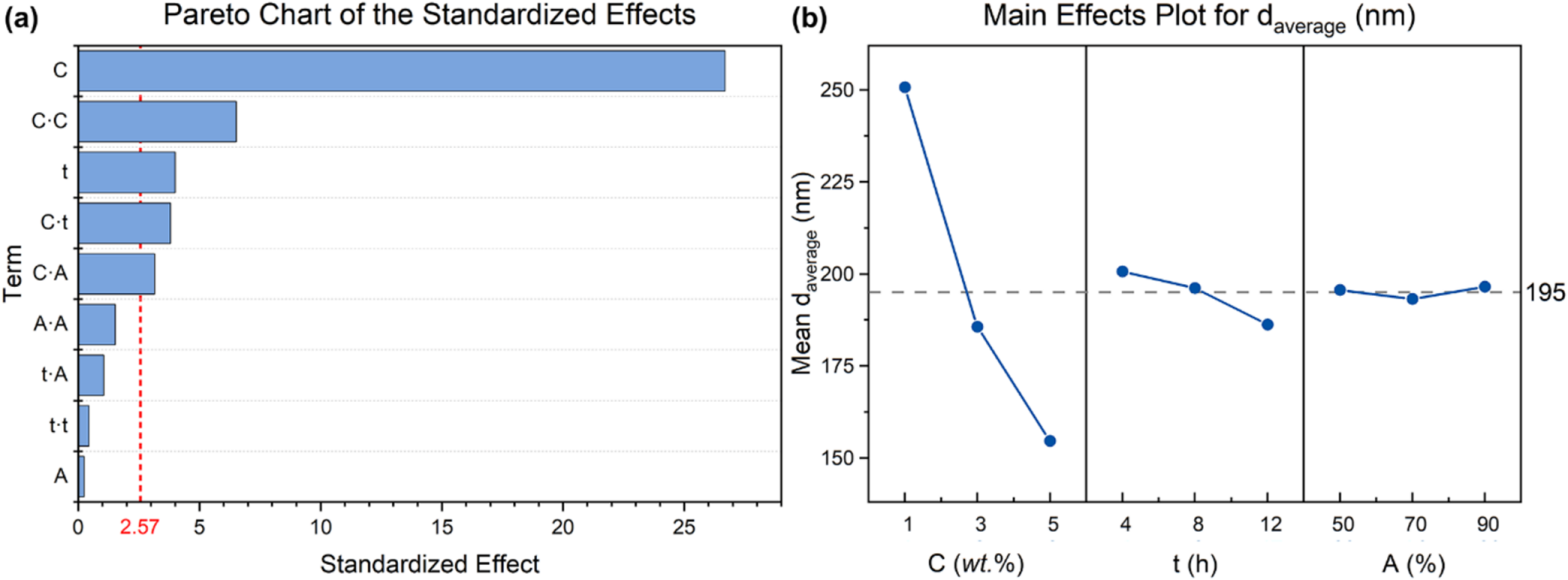
(a) Pareto chart of the standardized effects (with threshold statistical significance indicated as a dotted red line) and (b) main effects plot for average particle size (the mean value of *d*_average_ = 195 nm is also reported as a dashed gray line).

Clearly, the most significant parameter influencing the average LNP size is the concentration of the lignin-in-water suspension, as made evident by the magnitude of the bar associated with this variable (Figure 1a). On the contrary, little to no influence on *d*_average_ is registered for the processing time and sonication amplitude, respectively. In particular, a sharp nearly linear decreasing trend of lignin average particle size is observed at increasing suspension concentration, with *d*_average_ varying from 250 nm down to 155 nm for suspension concentrations of 1 and 5 wt %, respectively. Notably, a similar effect on LNP molecular weight was recorded, as outlined by GPC results (Table S3). Specifically, when considering the mean value of *M*_n_ across different levels of lignin concentration, the number-average molecular weight was found to progressively decrease from ∼1640 g/mol (for raw Indulin AT) to ∼1540 g/mol (for *C* = 1 wt %) to ∼1140 g/mol (for *C* = 3 wt %), down to ∼1065 g/mol (for *C* = 5 wt %) (Table S3). This indicates that an ultrasonic irradiation-induced size reduction occurred at both colloidal and molecular levels. Instead, a minor decrease in *d*_average_ is observed when the processing time is increased, and negligible changes are found upon increased processing amplitude (Figure 1b). These findings can be explained by the fact that at the early stages of the ultrasonication process, volume pulverization may be the dominant lignin size-reduction mechanism. Conversely, as the processing time increases, LNPs become progressively smaller, and their dimension cannot be further decreased by cavitation microjets and shockwaves, but only by mechanical abrasion and erosion.^31^ As surface erosion is a consequence of particles impacting with each other, it is expected that ultrasonic energy provided to a more packed colloidal system is more likely to promote efficient collisions among particles, thus inducing their effective size reduction, rather than to dissipate in the dispersing medium.

Based on these observations, the possibility of achieving even smaller LNP dimensions by further increasing the nominal lignin-in-water suspension concentration was gauged. Accordingly, two additional levels of suspension concentration were considered, lying out of the investigation range of the model (i.e., 10 and 20 wt %), and two additional experiments were performed by setting the values for the other two factors to their respective middle level—namely, processing time of 8 h and sonication amplitude of 70% (experiment 10–8–70: *C* = 10 wt %, *t* = 8 h, *A* = 70%; experiment 20–8–70: *C* = 20 wt %, *t* = 8 h, *A* = 70%). Very remarkably, in these conditions, nominal lignin-in-water concentrations of 10 and 20 wt % led to *d*_average_ values of 96 ± 5 and 95 ± 5 nm, respectively, as evidenced by DLS analysis (Table S1). This trend appears particularly promising when a prospective industrial upscale of the lignin ultrasonication process is considered. Indeed, 10–8–70 and 20–8–70 samples exhibited comparable chemical–physical properties in terms of the hydrodynamic dimension as well as morphology, chemical functionalities, and colloidal yield (see Table S1 for discussion of the other responses). These results underscore the versatility of ultrasonication, which enables to reproducibly obtain LNPs as small as ∼100 nm by operating at high lignin concentrations, while allowing wide leeway to optimally select the intensity and duration of the treatment based on desired outcomes in nanoparticle characteristics. This entails simplified downstream processing, and, ultimately, improved sustainability and overall efficiency.

^31^P NMR spectroscopy was used to assess the modifications potentially occurring in the chemical structure of lignin as a consequence of ultrasonic treatment. When liquid media undergo strong ultrasonic irradiation, the collapse of microbubbles generates extremely high local temperature and pressure conditions that may promote the formation of highly reactive species.^34–38^ In the case of an aqueous lignin suspension, acoustic cavitation not only favors the homolysis of water, leading to the generation of H· and ·OH radicals, but can also induce the homolytic scission of lignin macromolecular chains, resulting in radical pairs or monomers polymerization.^11,39^ Gilca et al. identified two main reaction patterns causing side-chain cleavage/depolymerization and oxidative coupling/polymerization, respectively, and hypothesized that one mechanism could be favored over the other via modulation of ultrasonic irradiation time and/or amplitude, thus potentially tuning the structural and compositional modifications of lignin.^11^

In the present work, the DoE analysis identified the lignin concentration as the only factor having a statistically significant effect on the total hydroxyl functionalities (Figure S1). This finding is consistent with ^31^P NMR data, since all of the experiments at the lowest *C* level (1 wt %) resulted in a slight increase in the amount of aliphatic OH compared to raw Indulin AT, while the opposite trend was registered for LNPs recovered from more concentrated suspensions (Figure 2 and Table S1). In addition, a decrease in the content of aromatic OH with respect to the untreated parent material was noted in all cases (Figure 2 and Table S1). To shed light on the chemical reactions taking place during the ultrasonic irradiation, these observations should be coupled with GPC results, which recorded progressively lower values of number-average molecular weight (*M_n_*) at increasing lignin concentration, accompanied by higher values of dispersity (*Đ*_GPC_) irrespective of the process conditions (Table S3). This suggests that the hydroxyl and superoxide radical species generated by the acoustic cavitation-induced homolysis of water promoted the occurrence of oxidative processes, overall leading to the cleavage of interunit linkages within the lignin structure (e.g., β-O-4, β-β, β-5). Ultimately, this leads to lignin depolymerization and degradation into smaller molecules. In such conditions, aromatic hydroxyl groups tend to readily take part as reactants in chemical processes like radical oxidation, as well as condensation and cross-linking among low-molecular weight fragments, resulting in an overall decrease in the number of phenolic functionalities. As for aliphatic hydroxyl groups, a concentration-dependent response was observed in this case, likely stemming from lower lignin content and higher energy dissipation in diluted systems. Conversely, multiple cleavage and recombination reactions of alkyl side chains are favored in more concentrated suspensions, yielding LNPs with decreased number-average molecular weight, as well as fewer hydroxyl functionalities. Finally, the DoE analysis evidenced the quadratic effects of sonication amplitude and lignin-in-water suspension concentration as the most significant factors influencing the carboxyl functionalities during the ultrasonication process, although no clear trends could be highlighted (Figures 2, S2 and Table S1).

**Figure 2:**
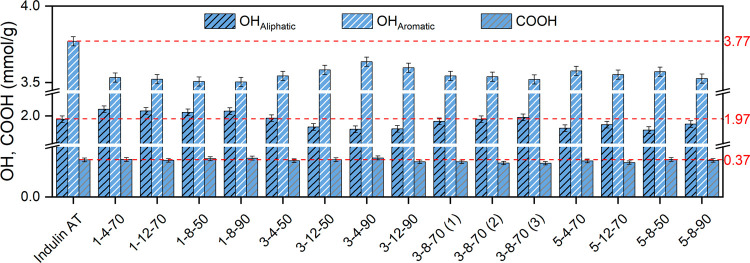
Concentration of aliphatic hydroxyl (OH_Aliphatic_), aromatic hydroxyl (OH_Aromatic_), and carboxyl (COOH) groups in pristine and ultrasound-treated lignin samples. Measured values for raw Indulin AT (i.e., 1.97, 3.77, and 0.37 mmol/g for OH_Aliphatic_, OH_Aromatic_, and COOH groups, respectively) are also reported as references (dashed red lines).

As widely acknowledged in the literature, one of the main drawbacks of obtaining LNPs via ultrasonication is related to the irregular morphology typically exhibited by the resulting particles.^2^ With the aim of employing ultrasound-treated LNPs as nanoscale fillers for the production of biobased nanocomposites with enhanced mechanical properties, their shape becomes one of the most important parameters influencing the nanostructure and the performance of the resulting material.^40^ Particles with jagged edges and sharp corners limit interfacial adhesion between the continuous phase and the dispersed phase, causing detachment and nanovoid formation during mechanical loading, which may lead to stress concentration and premature failure of the composite material.^40^ Conversely, particles with a smoother and more rounded profile are more easily incorporated into a host matrix, fostering a more effective stress transfer and a more uniform strain distribution, which is crucial for ensuring structural integrity and mechanical performance of the composite material.^40^ Therefore, in the present work, the possibility of controlling the shape of lignin particles by tuning the process parameters was investigated. Specifically, this issue was addressed by quantifying the morphological regularity through a property defined as “average particle shape circularity” (Π, calculated according to eq 3), which was taken as a two-dimensional indicator of the sphericity of ultrasound-treated LNPs. As evidenced by TEM images (Figure 3), progressively more roundish particles could be obtained by increasing the intensity of the treatment. Interestingly, perfectly circular profiles could be formed in all of the experiments conducted at the highest level of sonication amplitude (90%), likely suggesting that this process parameter could play a pivotal part in particle shaping during ultrasonic irradiation. This qualitative information was converted into a quantitative one by calculating the average particle shape circularity for each experiment with the purpose of numerically assessing how the morphology changed depending on the process conditions. These visual observations found validation in the statistical model, which identified sonication amplitude and lignin concentration as the only factors having a significant effect on Π, with the former being predominant over the latter (Figure S3). In particular, when considering the mean value of Π across different levels of sonication amplitude, the average particle shape circularity progressively increased from 0.70 (for *A* = 50%) to 0.84 (for *A* = 70%), up to 0.91 (for *A* = 90%) (Table S1 and Figure S3).

**Figure 3:**
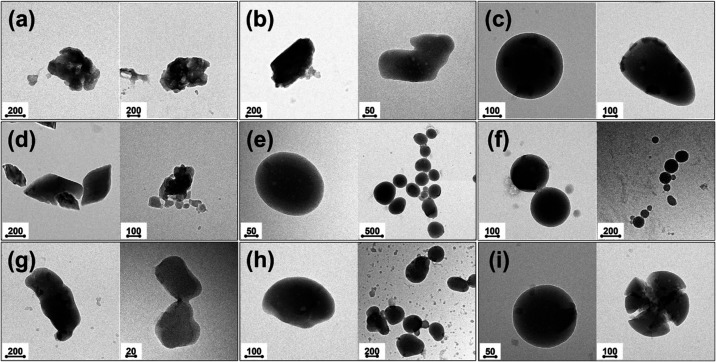
TEM images of aqueous LNP suspensions concentrated at 1 wt % (a–c), 3 wt % (d–f), and 5 wt % (g–i) lignin and sonicated at 50% (a, d, and g), 70% (b, e, and h), and 90% (c, f, and i) amplitude. The scale bars are expressed in nm.

This behavior can be explained by considering that the size-reduction process of LNPs is likely to proceed by surface erosion phenomena when LNPs reach a certain critical dimension, in line with the previous discussion. When ultrasonic irradiation is stronger, particles impact more frequently with each other and their edges are progressively smoothened and rounded by mechanical abrasion and erosion. It is worth highlighting that, among all of the experiments conducted at 90% sonication amplitude, conditions 3–4–90 (Figure 3f, left) and 3–12–90 (Figure 3f, right) yielded only LNPs with a purely spherical shape, while irregular nanoparticles could also be spotted when the lignin concentration was different than 3 wt %. Specifically, for *C* = 1 wt % (Figure 3a–c), oblong contours were found, suggesting that the colloidal system was too diluted and that part of the ultrasonic energy provided to it dissipated in the dispersing medium; for *C* = 5 wt % (Figure 3g–i), shattered particles were noticed, indicating that the aqueous suspension was too concentrated, and that LNPs fragmented due to repeated violent impacts with each other. To the authors’ knowledge, these findings represent the first documented instance of spherical LNPs obtained upon straightforward ultrasonic irradiation of suspensions of raw kraft lignin in water, using no chemicals capable of inducing pH/solvent shifting or self-assembly phenomena. These observations shed light on the underlying correlations between key process variables and the morphology of ultrasound-treated LNPs, making a pioneering contribution to the understanding of previously unexplored (and unexploited) features of this physical treatment.

From an environmental and process-cost point of view, the main drawback of obtaining nanolignin via ultrasonication is related to the requirement of freeze-drying for recovering the resulting particles. Freeze-drying was reported to be the most energy-demanding unit operation in ultrasound-assisted LNP production, and to be responsible for ∼85% of the average environmental impacts generated throughout the process.^41^ Within this framework, using ultrasound-treated lignin nanoparticle aqueous suspensions directly for dissolving water-soluble polymers represents a promising strategy to develop lignin-based nanocomposite materials while avoiding freeze-drying and its negative effects on the environment. To this end, quantification of the actual amount of lignin that remains dispersed and usable post-treatment would be crucial for ensuring the correct filler loading in the polymeric blends. To meet this challenge, the mass fraction of suspended LNPs in water after ultrasonication (*x*_lig,sus_) was taken as a colloidal yield of the process and was investigated as an additional target system response. As shown in Figure 4, *x*_lig,sus_ was found to strongly depend on the duration and the power intensity of the ultrasonic treatment, with no effect given by the initial suspension concentration. Specifically, regardless of the nominal starting lignin concentration, lignin-in-water suspensions subjected to the same combination of ultrasound-treatment power and processing time resulted in roughly the same colloidal yield. Furthermore, it was observed that the higher the level of factors *t* and *A*, the higher the value of *x*_lig,sus_ (Table S1). This trend can be explained by considering that a more intense and prolonged ultrasonic irradiation process on the colloidal system is expected to yield a higher fraction of solid agglomerates that undergo volume pulverization and break into single particles. This in turn will lead to particles of sizes smaller than the critical dimension that allow them to remain stably suspended in the dispersing medium, ultimately resulting in improved colloidal yield.

**Figure 4:**
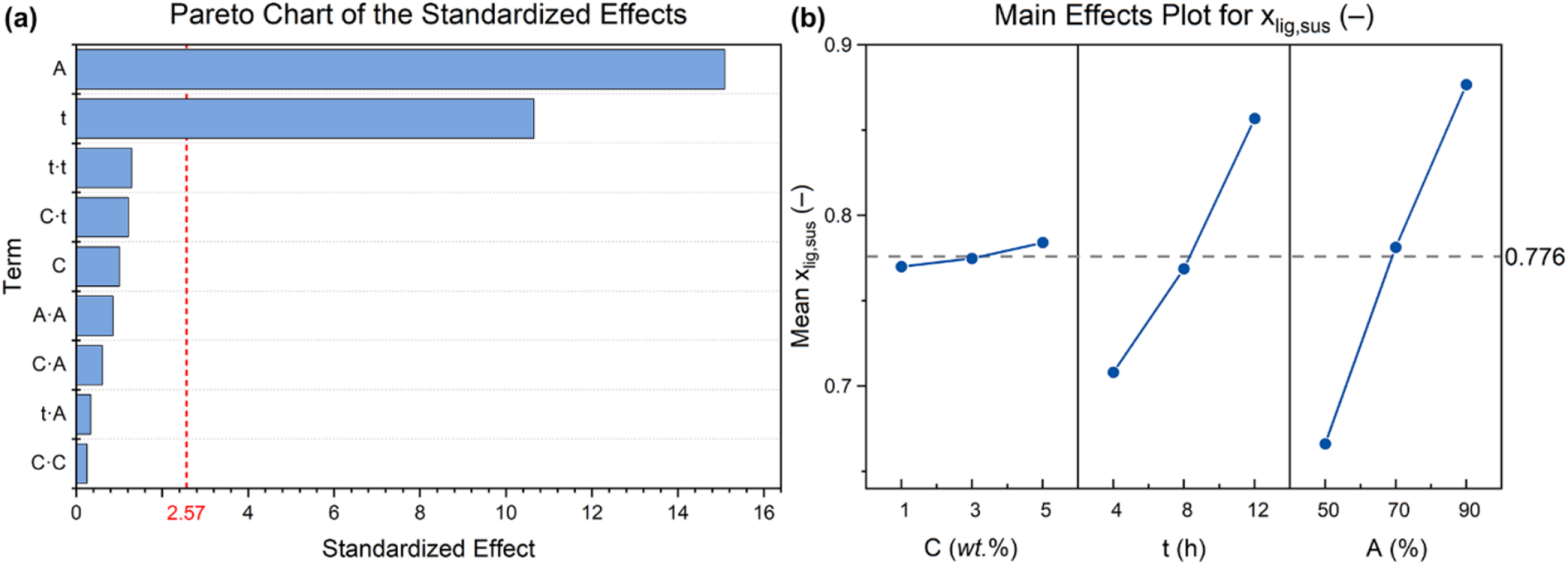
(a) Pareto chart of the standardized effects (with threshold statistical significance indicated as a dotted red line) and (b) main effects plot for mass fraction of suspended lignin particles (the mean value of *x*_lig,sus_ = 0.776 is also reported as a dashed gray line).

In light of all of the results obtained from this comprehensive statistical analysis on LNP formation by ultrasonic treatment in aqueous media, noteworthy conclusions can be drawn. In this work, we demonstrated the possibility of producing readily usable water suspensions of chemically modified spherical LNPs smaller than 100 nm exhibiting excellent colloidal yield. This could be achieved through an easily implementable technique relying solely on the action of mechanical forces, with no use of solvents and/or other chemicals. Interestingly, recent early-stage life-cycle assessment studies on different LNP production methods (solvent/pH shifting and ultrasonication) have shown that the largest negative environmental impact in the nanolignin formation process (quantified in terms of global warming potential, GWP) is contributed by solvent recovery and freeze-drying, respectively.^41,42^ Based on these preliminary outcomes, our proposed protocol (production of LNP suspensions in water by ultrasounds and their direct use as processing media for water-soluble polymers) appears as an environment-friendly strategy to develop lignin-based nanocomposite materials while avoiding the environmental impacts associated with the requirement for solvents and/or freeze-drying.

### 3.2 PVA-Based Films Characterization

Based on the outcomes of the process study carried out on the ultrasound-assisted LNP formation in water suspension, PVA-based films incorporating increasing loads (5, 10 and 20% (w/w)) of either pristine or suitably ultrasound-treated lignin were prepared to evaluate the effect of tailored lignin (nano)­particle characteristics on the chemical, morphological, thermal, and mechanical properties of the resulting (nano)­composite systems.

The level of dispersion of (nano)­lignin within the PVA host matrix at increasing filler loading was assessed by means of SEM images of cryofractured samples (Figure 5). At increasing untreated parent lignin content, progressively rougher fracture surfaces were observed with evident agglomerates and clusters unevenly distributed within the continuous PVA phase (Figure 5a–c). These defects limit interfacial adhesion between lignin and PVA, and act as stress concentration points during mechanical loading, causing cracks initiation and propagation, eventually leading to premature failure of the material. Conversely, thanks to the smaller dimension, more regular shape, and lower molecular weight of LNPs, nanocomposite systems exhibited noticeably smoother and more homogeneous fracture surfaces as a result of improved matrix–filler compatibility and excellent dispersed-phase distribution (Figure 5d–f).

**Figure 5:**
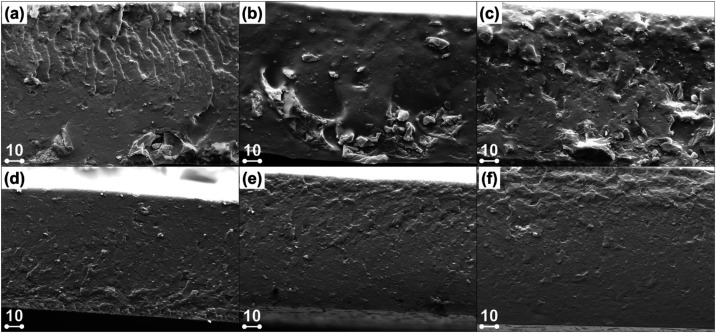
SEM images of PVA-based films incorporating 5 wt % (a, d), 10 wt % (b, e), and 20 wt % (c, d) of either pristine (a–c) or ultrasound-treated (d–f) lignin particles. The scale bars are expressed in μm.

**Figure 6:**
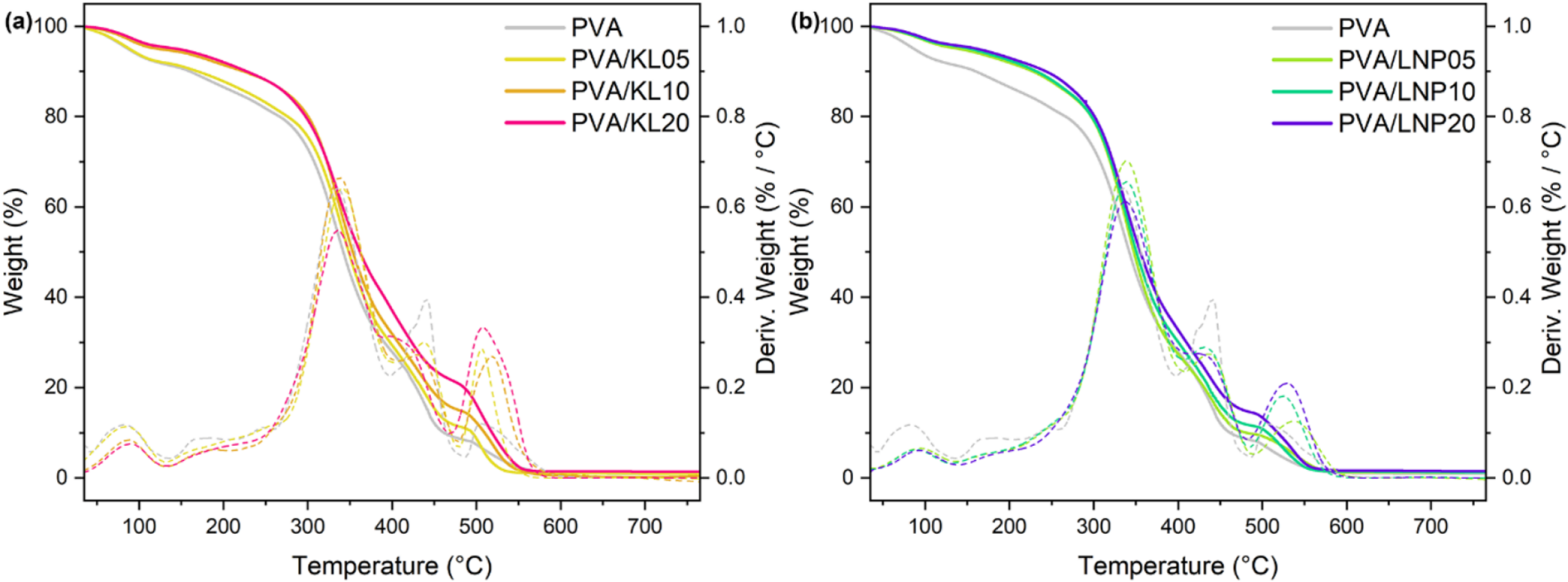
Thermogravimetric curves (solid lines) and derivative thermogravimetric curves (dashed lines) for (a) PVA/lignin and (b) PVA/LNPs systems. TG and DTG curves for neat PVA are reported as reference (gray lines).

The thermooxidative stability and thermal decomposition of different PVA-based (nano)­composite systems were investigated by means of TGA performed in air (Figure 6 and Table S5). The neat PVA material exhibited the first thermal degradation event in the 75–125 °C temperature range, associated with the evaporation of adsorbed water. Due to this, a first mass loss of 5% occurred at a relatively low temperature for the unfilled material (i.e., *T*_5% mass‑loss_ = 87 °C). Below 150 °C, both composite and nanocomposite systems exhibited higher thermooxidative stability than pure PVA, likely indicating that the presence of lignin reduces the amount of water physically adsorbed from the environment.^43,44^ Notably, when comparing the formulations with the lowest filler loading (PVA/KL05), little variation in the temperature corresponding to a mass loss of 5% was recorded in the presence of the untreated parent material (i.e., *T*_5% mass‑loss_ = 89 °C), while a more noticeable enhancement in thermal stability was found in the case of nanolignin-based systems (PVA/LNP05), for which a *T*_5% mass‑loss_ of 140 °C was registered. The second thermal degradation event was found between 150 and 450 °C, where all of the materials exhibited a ∼60% mass loss, likely due to decomposition of the PVA backbone into carbon monoxide, carbon dioxide, and methane, as well as to cleavage of α- and β-aryl–alkyl-ether bonds within the lignin macromolecular structure.^45–47^ Within this temperature range, all of the systems lost 10% of their respective initial weight. In particular, values of *T*_10% mass‑loss_ = 170 °C (very close to 160 °C, observed for the unfilled material), 224, and 228 °C were obtained for PVA/KL05, PVA/KL10, and PVA/KL20, respectively. As opposed to this, *T*_10% mass‑loss_ = 228, 232, and 244 °C were obtained for PVA/LNP05, PVA/LNP10, and PVA/LNP20, respectively. A third mass-loss event was observed between 450 and 600 °C, ascribable to the cleavage of intramolecular C–C bonds within lignin phenylpropane units, leading to the removal of small terminal groups (e.g., alcohols, phenolics, aldehydes, and acids), to the release of gaseous species, and to the generation of charcoal.^46,47^ Based on these observations, lignin was found to impart enhanced thermooxidative stability to the PVA–lignin (nano)­composites as a result of a twofold action: on the one hand, its highly aromatic structure can favor the formation of a highly refractory charcoal protective layer, slowing down the thermal degradation process; on the other hand, the abundance of phenolic hydroxyl groups contributes to a favorable radical scavenging effect, partially inhibiting thermal oxidation.^48,49^ Remarkably, PVA/LNPs nanocomposites exhibited a higher thermooxidative stability than the corresponding PVA/lignin systems for the same filler loading. This behavior can be attributed to the smaller dimension and more regular shape of ultrasound-treated lignin nanoparticles, which may foster stronger matrix–filler interactions and a more homogeneous distribution of nanolignin within the PVA matrix, ultimately resulting in improved functional (thermooxidative) response.

To investigate the effect of the ultrasonic treatment carried out on lignin on the mechanical properties of the resulting PVA-based (nano)­composites, uniaxial tensile tests were performed on systems incorporating 5% (w/w), 10% (w/w), and 20% (w/w) of either pristine or ultrasound-treated lignin (Figure 7). The incorporation of lignin into PVA resulted in increased stiffness for a higher lignin loading. Compared to the reference unfilled PVA material (tensile elastic modulus *E_t_* = 41 MPa), progressively higher values of *E_t_* were measured for both composite and nanocomposite systems as the lignin content increased (up to 181 MPa for PVA/KL20, and 135 MPa for PVA/LNP20) as a result of the contribution provided by the rigid aromatic structure of lignin to the mechanical response of the (nano)­composites. Interestingly, the formulations containing pristine lignin (PVA/KL) exhibited systematically higher modulus, lower strength, and larger statistical variability (viz., larger standard deviation) with respect to the nanocomposite PVA/LNP systems at the same filler loading. In particular, when incorporated into the host matrix, untreated lignin particles tended to aggregate into large, randomly shaped, high-molecular-weight clusters, which severely hinder the local mobility of PVA backbone chains, leading to increased overall rigidity and higher *E_t_* values. Conversely, thanks to their nanometric dimension, extremely regular morphology, and lower molecular weight, LNPs were evenly distributed within the continuous phase, allowing PVA to retain some of its inherent flexibility. Additionally, values of σ_u_ = 13, 15, and 11 MPa were obtained for PVA/KL05, PVA/KL10, and PVA/KL20, respectively, as opposed to σ_u_ = 15, 17, and 15 MPa were obtained for PVA/LNP05, PVA/LNP10, and PVA/LNP20, respectively. It is worth highlighting that in the case of the highest filler loading (20% w/w), untreated lignin led to a notable decrease in σ_u_ with respect to the neat unfilled PVA material (σ_u_ = 13 ± 1.3 MPa), likely due to the poor interfacial adhesion and stress transfer between the continuous and dispersed phases in this case. Conversely, a systematic 15–30% increase in σ_u_ was registered upon incorporation of ultrasound-treated nanoparticles, thanks to the more effective matrix–filler interactions and (nano)­lignin dispersion. Remarkably, while a similar average response was observed in both composite (PVA/KL) and nanocomposite (PVA/LNP) systems at low-to-moderate filler loadings (5% and 10%) in terms of ε_b_ (∼155–149%), striking differences were found at the highest extent of lignin incorporation (20% w/w). In particular, in the case of nanocomposite systems (PVA/LNP20), a ε_b_ value of ∼100% was recorded, as opposed to ε_b_ = ∼35% for PVA/KL20 composites, where a brittle-like response could be observed. This behavior can be explained by the strong tendency of untreated lignin particles to form microsized irregularly shaped aggregates within the polymeric matrix, which can act as defects causing crack initiation and propagation during mechanical loading, ultimately leading to premature failure of the material during deformation. On the contrary, the fine control over size, shape, and morphology of LNPs provided by the ultrasonic treatment, coupled with their excellent dispersion within the PVA matrix, allows the retention of acceptable mechanical properties even at relatively high filler loadings.

**Figure 7:**
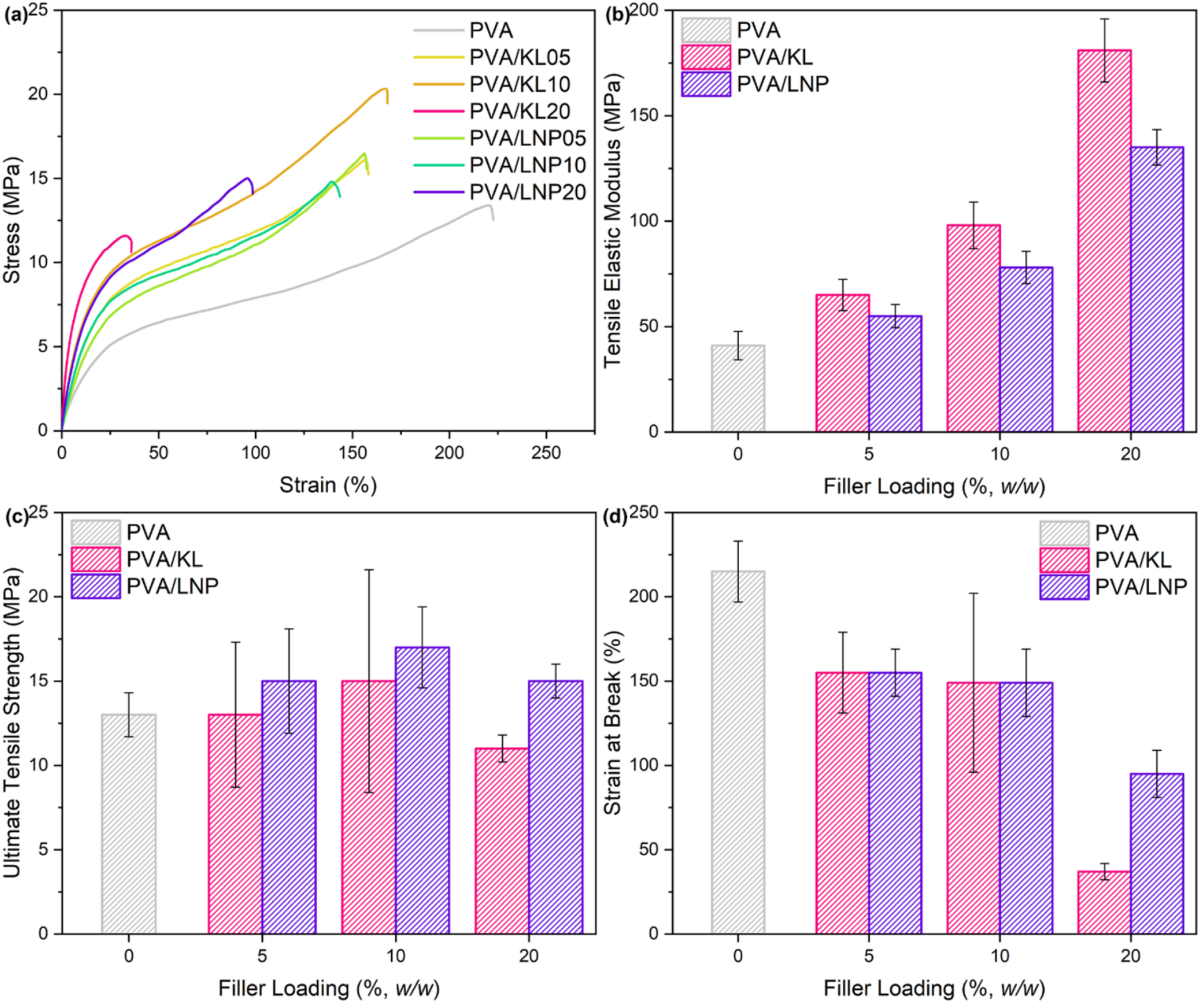
(a) Representative strain–stress curves of the PVA, PVA/lignin, and PVA/LNPs systems. Mean values of (b) tensile elastic modulus (*E_t_*), (c) ultimate tensile strength (σ_u_), and (d) strain at break (ε_b_) for composite and nanocomposite systems at varying filler loading. The neat unfilled PVA material is reported as a reference (gray line/bars). Error bars indicate the standard deviation of at least five measurements on different specimens.

## 4 Conclusions

In this work, a DoE methodology was used to study the lignin ultrasonication process so as to establish qualitative and quantitative cause–effect relationships between relevant operating variables (i.e., lignin concentration, processing time, and sonication amplitude) and target characteristics of the obtained nanoparticles. Lignin concentration in the suspension was found to play a key role in determining the hydrodynamic dimensions and chemical functionalities of LNPs. Additionally, by appropriately tuning ultrasonic irradiation amplitude and lignin-in-water suspension concentration (the only factors having a statistically significant effect on particle morphology), nanospheres with perfectly circular profiles were observed to form. This represents the first successful attempt at obtaining spherical LNPs through straightforward ultrasonic irradiation of raw kraft lignin in water, using no chemicals capable of inducing pH/solvent shifting or self-assembly phenomena. Finally, the mass fraction of suspended LNPs in water—taken as a colloidal yield—was shown to be mainly affected by the intensity and duration of the treatment.

Based on the outcomes of the process study carried out on the ultrasound-assisted LNP formation in water suspension, PVA-based films incorporating increasing loads (up to 20% (w/w)) of either pristine or suitably ultrasound-treated lignin were prepared to evaluate the effect of tailored lignin (nano)­particle characteristics on the chemical, morphological, thermal, and mechanical properties of the resulting (nano)­composite systems. The observed trends were associated with the smaller dimension and more regular shape of ultrasound-treated lignin nanoparticles, which fostered a higher level of dispersion of nanolignin within PVA together with a more effective matrix–filler interaction and stress transfer, ultimately yielding nanocomposite materials with improved thermal response and enhanced mechanical properties.

This work provides key insights into the production of LNPs via ultrasonication, demonstrating that precise control over their size, morphology, and chemical functionalities can be predictively attained by optimal tuning of easily accessible process parameters. These findings pave the path toward a more efficient production of lignin nanoparticles and their use as macromolecular fillers in biobased polymeric nanocomposites for advanced and sustainable manufacturing.

## Supplementary Material



## Notes

Measured values of *d*_average_, OH, COOH, Π, and *x*_lig,sus_ for pristine and ultrasound-treated lignins; regression coefficients and *R* ^2^ of the fitting models; measured values of *T*_g_, PDI_DLS_, *M_n_*, *M*_w_, and *Đ*_GPC_ for pristine and ultrasound-treated lignins; Pareto charts of the standardized effects and main effects plots for OH, COOH, and Π; SEM images of untreated Indulin AT; SEM images of cryofractured cross sections of unfilled PVA films; FTIR spectra of PVA-based films; characteristic transition temperatures of PVA-based films; and characteristic mass-loss temperatures of PVA-based films (PDF)

## Abbreviations

ATR: attenuated total reflectance
BBD: Box–Behnken design
DLS: dynamic light scattering
DoE: Design of Experiments
DSC: differential scanning calorimetry
FTIR: Fourier transform infrared
GPC: gel permeation chromatography
KL: kraft lignin
LNP: lignin nanoparticle
NMR: nuclear magnetic resonance
PDI: polydispersity index
PVA: poly­(vinyl alcohol)
SEM: scanning electron microscopy
TEM: transmission electron microscopy
TGA: thermogravimetric analysis
