# Supplementary material for: Nanolignin by Ultrasonication: Tuning the Process for Tailored Materials Characteristics
Source: ACS Omega. 2025 Jun 21;10(25):27585–97. doi: 10.1021/acsomega.5c03665 (PMC12224091; doi:10.1021/acsomega.5c03665)
Supplement: Supplementary file 1 [file ao5c03665_si_001.pdf]

Supporting Information

Nanolignin by ultrasonication: tuning the process for tailored materials characteristics

Eleonora Ruffini[a], *Emanuela Bellinetto*[a], *Stefano Turri*[a], *Gianmarco Griffini*[a*]

[a]Department of Chemistry, Materials and Chemical Engineering "Giulio Natta", Politecnico di Milano, Piazza Leonardo da Vinci 32, 20133 Milano, Italy.

*Corresponding author: gianmarco.griffini@polimi.it

Table of Contents

Pristine and ultrasound-treated lignins characterization S3

Box-Behnken design on lignin ultrasonication process S3

Results of other analyses performed on pristine and ultrasound-treated lignins S8

PVA-based films characterization S10

Chemical and morphological characterization of PVA-based films S10

Thermal and thermo-oxidative characterization of PVA-based films S12

References S14

Index of Figures

Figure S1 Pareto chart of the standardized effects and main effects plot for total OH content. ...S6

Figure S2 Pareto chart of the standardized effects and main effects plot for COOH content. S6

Figure S3 Pareto chart of the standardized effects and main effects plot for Π. S7

Figure S4 SEM images of untreated Indulin AT. S9

Figure S5 FTIR spectra for PVA-based films. S10

Figure S6 SEM images of cryo-fractured cross-section of unfilled-PVA film. S11

Index of Tables

Table S1 Measured values of $d_{average}$, OH, COOH, Π, and $x_{lig,sus}$ of lignin samples. S3

Table S2 Regression coefficients and R^2 of the fitting models. S5

Table S3 Measured values of T_g, PDI_{DLS}, M_n, M_w, and $Đ_{GPC}$ of lignin samples. S8

Table S4 Characteristic transition temperatures of PVA-based films. S12

Table S5 Characteristic mass-loss temperatures of PVA-based films. S13

Pristine and ultrasound-treated lignins characterization

Box-Behnken design on lignin ultrasonication process

Table S1 Measured average particle size ($d_{average}$), concentration of total hydroxyl groups (OH), concentration of carboxyl groups (COOH), average particle circularity (Π), and mass fraction of suspended lignin particles in water ($x_{lig,sus}$) for pristine and ultrasound-treated (used to either fitting or validating the models) lignin samples.

Sample[1]	$d_{average}$ (nm)	OH (mmol/g)	COOH (mmol/g)	Π (–)	$x_{lig,sus}$ (–)
1-4-70	250 ± 12	5.59 ± 0.03	0.37 ± 0.02	0.63 ± 0.16	0.72 ± 0.04
5-4-70	171 ± 8	5.47 ± 0.03	0.36 ± 0.02	0.90 ± 0.03	0.71 ± 0.04
1-12-70	249 ± 12	5.57 ± 0.03	0.37 ± 0.02	0.71 ± 0.13	0.84 ± 0.04
5-12-70	131 ± 6	5.47 ± 0.03	0.34 ± 0.02	0.91 ± 0.03	0.88 ± 0.04
1-8-50	246 ± 12	5.54 ± 0.03	0.38 ± 0.02	0.66 ± 0.07	0.64 ± 0.03
5-8-50	169 ± 8	5.44 ± 0.03	0.38 ± 0.02	0.75 ± 0.11	0.66 ± 0.03
1-8-90	258 ± 12	5.55 ± 0.03	0.39 ± 0.02	0.88 ± 0.06	0.88 ± 0.04
5-8-90	149 ± 8	5.45 ± 0.03	0.37 ± 0.02	0.90 ± 0.04	0.88 ± 0.04
3-4-50	191 ± 10	5.52 ± 0.03	0.36 ± 0.02	0.67 ± 0.12	0.60 ± 0.03
3-12-50	177 ± 8	5.48 ± 0.03	0.37 ± 0.02	0.71 ± 0.08	0.76 ± 0.04

3-4-90	191 ± 10	5.52 ± 0.03	0.39 ± 0.02	0.92 ± 0.01	0.80 ± 0.04
3-12-90	188 ± 9	5.48 ± 0.03	0.35 ± 0.02	0.95 ± 0.01	0.95 ± 0.05
3-8-70 (1)	183 ± 9	5.49 ± 0.03	0.35 ± 0.02	0.93 ± 0.01	0.80 ± 0.04
3-8-70 (2)	186 ± 9	5.51 ± 0.03	0.34 ± 0.02	0.92 ± 0.02	0.77 ± 0.04
3-8-70 (3)	183 ± 9	5.51 ± 0.03	0.34 ± 0.02	0.91 ± 0.04	0.75 ± 0.04
Indulin AT	–	5.74 ± 0.03	0.37 ± 0.02	–	–
2-6-60	207 ± 10	5.53 ± 0.03	0.32 ± 0.02	0.75 ± 0.05	0.70 ± 0.04
4-10-80	161 ± 8	5.47 ± 0.03	0.32 ± 0.02	0.95 ± 0.01	0.85 ± 0.04
10-8-70	96 ± 5	5.64 ± 0.03	0.31 ± 0.02	0.94 ± 0.02	0.79 ± 0.04
20-8-70	95 ± 5	5.64 ± 0.03	0.32 ± 0.02	0.94 ± 0.02	0.79 ± 0.04

[1]The samples are labelled as X-Y-ZZ, where "X" indicates the lignin concentration (*i.e.*, C (*wt.%*)); "Y" indicates the processing time (*i.e.*, t (h)); and "ZZ" indicates the sonication amplitude (*i.e.*, A (*%*)).

10-8-350 and 20-8-350 samples exhibited an equivalent amount of chemical functionalities (OH content of 5.64 ± 0.03 mmol/g, and COOH content of 0.32 ± 0.02 mmol/g), an extremely-regular nanoparticles morphology (average particle circularity of 0.94 ± 0.02), and a comparable suspended-lignin fraction with respect to each other ($x_{lig,sus}$ value of 0.79 ± 0.03) as well as to the suspensions subjected to the same combination of ultrasonication power and processing time, i.e., 3-8-350 (1), 3-8-350 (2), and 3-8-350 (3) (suspended-lignin fraction of 0.80 ± 0.04, 0.77 ± 0.03, and 0.75 ± 0.03, respectively), as predicted by the DoE analysis, which revealed that $x_{lig,sus}$ depends only on the intensity and duration of the treatment. These results evidence that, upon a proper selection of the process parameters, ultrasonication allows to efficiently produce spherical lignin nanoparticles as small as ~100 nm, without negatively affecting their chemical properties.

Table S2 Regression coefficients and coefficient of determination (R^2) of the fitting models for the prediction of average particle size ($d_{average}$), concentration of total hydroxyl groups (OH) and carboxyl groups (COOH), average particle circularity (Π), and mass fraction of suspended lignin particles in water ($x_{lig,sus}$) values at any experimental point within the investigation range.

Coefficient	$d_{average}$ (nm)	OH (mmol/g)	COOH (mmol/g)	Π (–)	$x_{lig,sus}$ (–)
β_0	+300.1	+5.5097	+0.5827	–0.761	+17.7
β_1	–26.19	–0.0454	–0.01591	+0.1950	–0.18
β_2	–0.212	–0.01921	+0.004040	+0.0748	+0.38
β_3	+0.68	+0.001067	–0.001259	+0.00471	+0.1824
β_{11}	+4.317	+0.00202	+0.003472	–0.01852	+0.064
β_{22}	–0.074	+0.000816	+0.000339	–0.00369	+0.0826
β_{33}	+0.000404	–0.000002	+0.000002	–0.000005	–0.000088
β_{12}	–1.212	+0.000954	–0.000260	–0.00221	+0.150
β_{13}	–0.0401	+0.000001	–0.000020	–0.000085	–0.00300
β_{23}	+0.00669	+0.000001	–0.000030	–0.000013	–0.00083
R^2	0.9938	0.9719	0.9712	0.9119	0.9858

The Pareto chart of the standardized effects and the main effects plot for total hydroxyl groups content (OH), carboxyl groups content (COOH), and average particle circularity (Π) are reported below (Figure S1, S2 and S3). The reference line for statistical significance (*i.e.*, dotted red line) on the Pareto charts indicates which effects are statistically significant at the 0.05 level with the

current model terms, and it is drawn at a value corresponding to the (1 – α / 2) quantile of a t-distribution with degrees of freedom equal to the degrees of freedom for the error term (*i.e.*, 5)[S1].

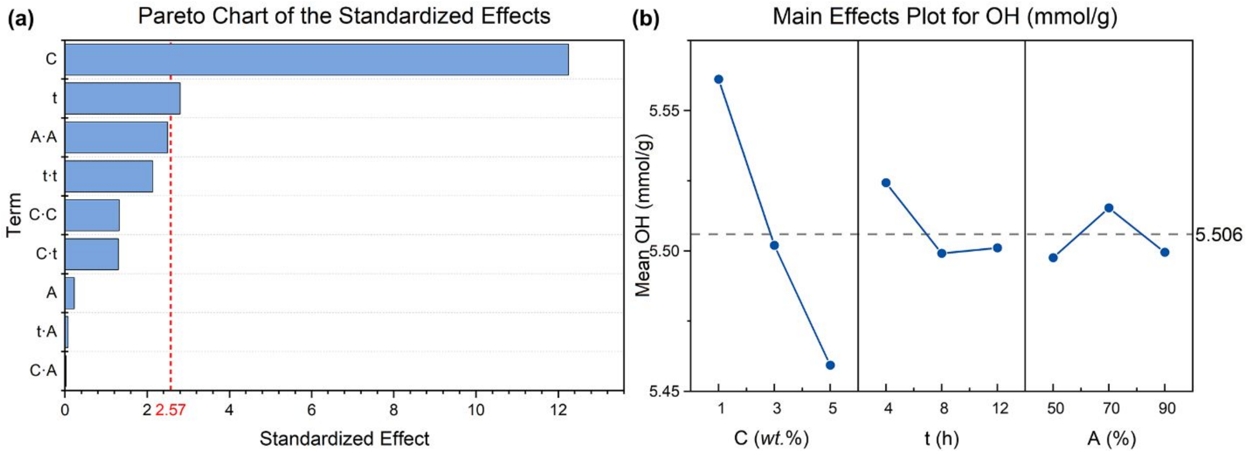

Figure S1 (a) Pareto chart of the standardized effects (with threshold statistical significance indicated as dotted red line) and (b) main effects plot for total hydroxyl groups content (the mean value of OH = 5.506 mmol/g is also reported as dashed grey line).

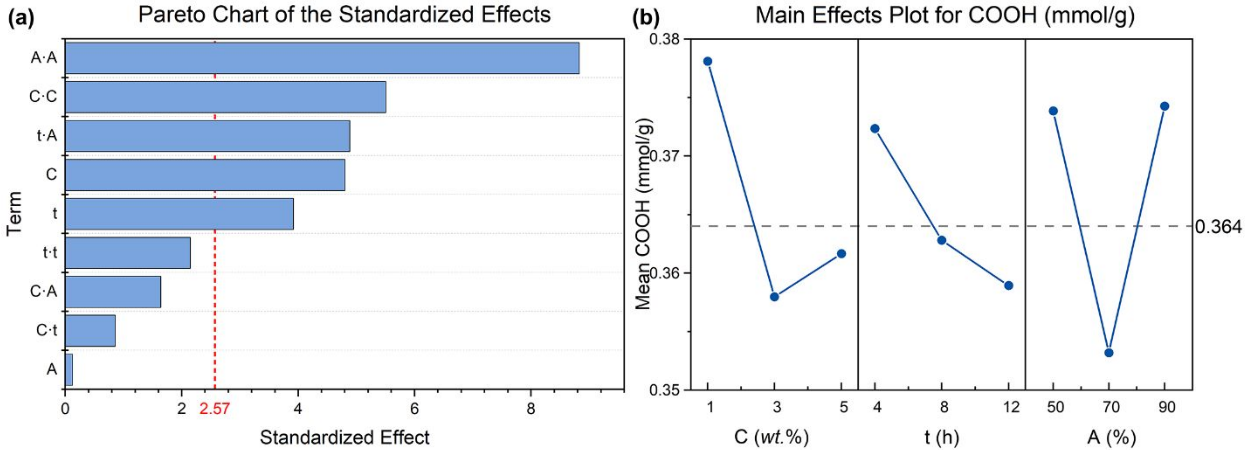

Figure S2 (a) Pareto chart of the standardized effects (with threshold statistical significance indicated as dotted red line) and (b) main effects plot for carboxyl groups content (the mean value of COOH = 0.364 mmol/g is reported as dashed grey line).

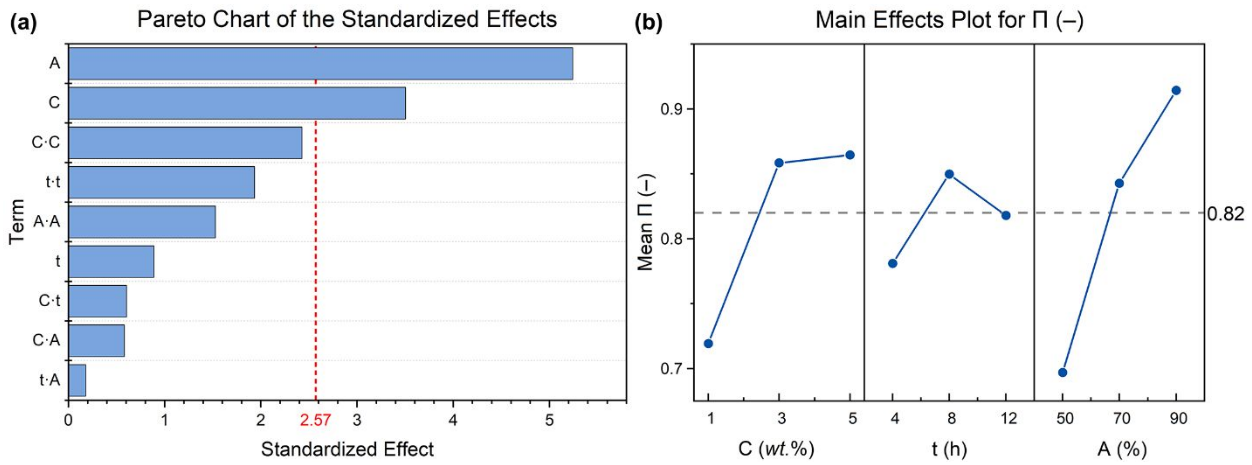

Figure S3 (a) Pareto chart of the standardized effects (with threshold statistical significance indicated as dotted red line) and (b) main effects plot for average particle circularity (the mean value of $\Pi = 0.82$ is also reported as dashed grey line).

Results of other analyses performed on pristine and ultrasound-treated lignins

Table S3 Measured glass transition temperature (T_g), nanoparticular polydispersity index (PDI_{DLS}), number-average molecular weight (M_n), wight-average molecular weight (M_w), and dispersity ($Đ_{GPC}$) for pristine and ultrasound-treated lignin samples.

Sample[1]	T_g (˚C)	PDI_{DLS} (–)	M_n (g/mol)	M_w (g/mol)	$Đ_{GPC}$ (–)
1-4-70	160	0.213	1500	5214	3.5
5-4-70	160	0.325	1060	3595	3.4
1-12-70	163	0.232	1333	4828	3.6
5-12-70	159	0.293	1020	3770	3.7
1-8-50	159	0.206	1650	6100	3.7
5-8-50	157	0.308	1065	3595	3.4
1-8-90	160	0.202	1680	6000	3.6
5-8-90	161	0.287	1110	3883	3.5
3-4-50	159	0.375	1288	4334	3.4
3-12-50	159	0.287	1248	4140	3.3
3-4-90	160	0.188	1122	3848	3.4
3-12-90	163	0.311	1123	4100	3.6
3-8-70 (1)	160	0.213	1080	4433	4.1
3-8-70 (2)	160	0.250	1050	4200	4.0
3-8-70 (3)	159	0.235	1070	4383	4.1
Indulin AT	160	–	1636	3842	2.3

[1]The samples are labelled as X-Y-ZZ, where "X" indicates the lignin concentration (*i.e.*, C (*wt.%*)); "Y" indicates the processing time (*i.e.*, t (h)); and "ZZ" indicates the sonication amplitude (*i.e.*, A (%)).

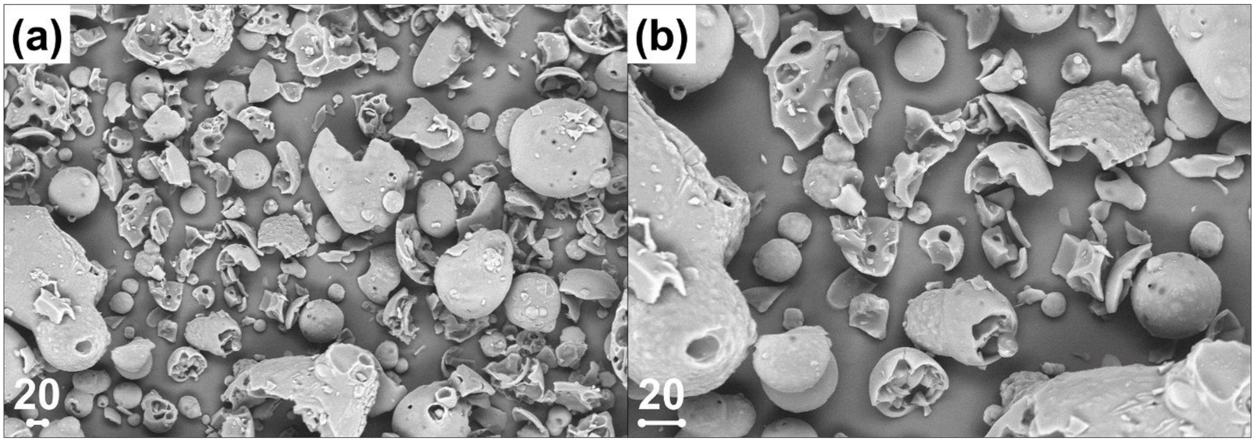

Figure S4 SEM images of untreated Indulin AT at (a) 500 × and (b) 1.0k × magnification. Scale-bars are expressed in μm.

PVA-based films characterization

Chemical and morphological characterization of PVA-based films

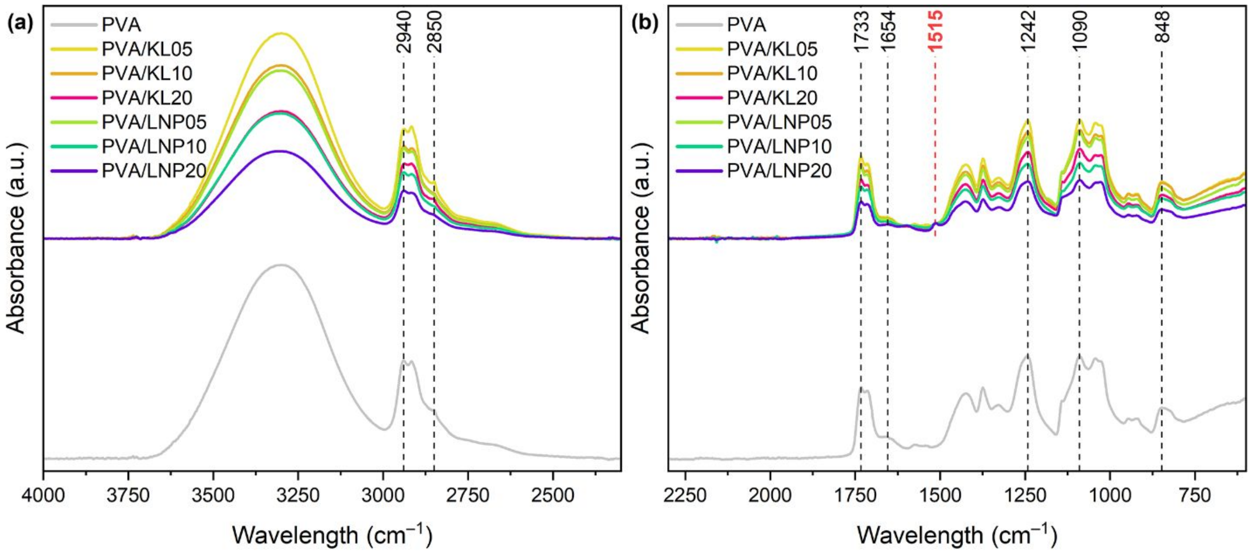

Figure S5 FTIR spectra for PVA/lignin and PVA/nanolignin systems (spectra were normalized using the peak at 1515 cm^{-1} as invariant band) in the (a) 4000–2300 cm^{-1} and (b) 2300–600 cm^{-1} wavelength ranges. The FTIR spectrum for neat PVA is reported as reference (grey line).

As shown in Figure S5, the O–H stretching vibration band is found to shift from 3311 cm^{-1} for pure PVA to 3300 cm^{-1} for all the lignin-based blends, indicating the occurrence of dipole-dipole attractive interactions (*viz.*, hydrogen bonding) between matrix and filler, as well as within PVA[S2–S4]. The peaks at 2940 and 2915 cm^{-1} were ascribed to C–H asymmetrical and symmetrical stretching of methyl CH_3, respectively, while the shoulder at 2850 cm^{-1} was assigned to C–H symmetrical stretching of methylene CH_2[S5,S6]. The signal at 1733 cm^{-1} was associated with C=O stretching of carbonyl functionalities (*i.e.*, unalcoholized acetate groups and oxidized hydroxyl

groups during PVA synthesis and processing), while the shoulder at 1654 cm^{-1} is attributable to O–H bending vibrations of physically-adsorbed water[S3,S7,S8]. The C–H bending vibration bands were detected at 1421, 1326, and 1242 cm^{-1}, with the latter being referrable to skeletal C–H wagging in PVA backbone chain[S9,S10]. The peaks at 1090 and 848 cm^{-1} were related to C–O and C–C stretching vibrations, respectively[S6,S9]. As for the two-phase systems, due to aromatic ring vibrations within lignin macromolecular structure, all the spectra exhibited a characteristic signal at 1515 cm^{-1} [S3,S11]. As expected, after normalization, all the spectra were observed to progressively shift to lower absorbance values as filler loading increased. More interestingly, when comparing the formulations at the same dispersed-phase content, nanocomposite systems showed systematically higher absorbed infrared radiation at 1515 cm^{-1} than composite ones (*e.g.*, the spectrum for PVA/LNP05 was shifted to lower absorbance values compared to the spectrum for PVA/KL05, and the same holds for higher lignin concentrations). This is likely due to the fact that, while LNPs are more prone to evenly distribute within the continuous phase, raw Indulin AT has a strong tendency to form coarse agglomerates randomly dispersed throughout the bulk of the material, resulting in concentration gradients and local sites where the actual filler loading is lower than the nominal one.

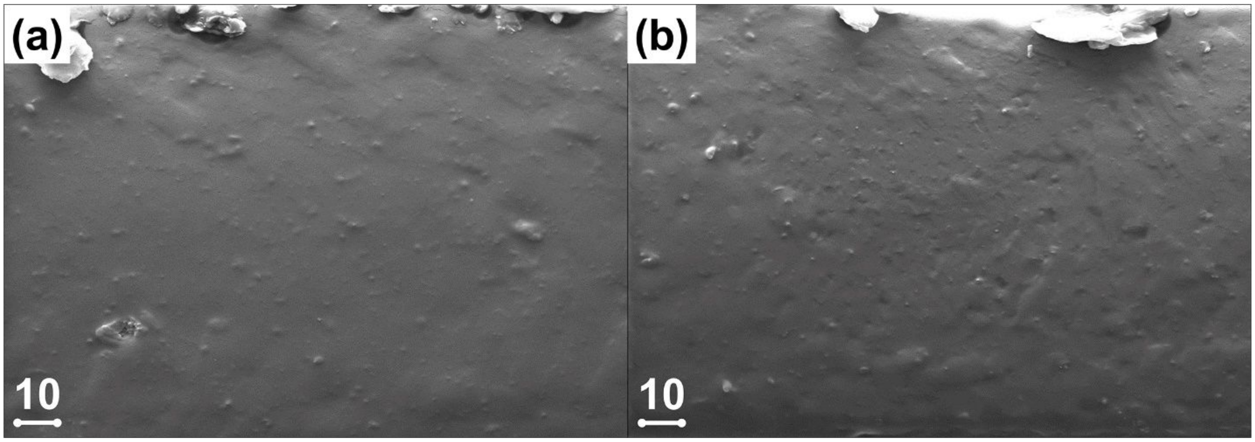

Figure S6 SEM images of cryo-fractured cross-section of unfilled-PVA film at 2.5k × magnification, and (a) 8.0 mm and (b) 7.5 mm working distance. Scale-bars are expressed in μm.

Thermal and thermo-oxidative characterization of PVA-based films

Table S4 Glass transition temperature (T_g), melting temperature (T_m), melting enthalpy (ΔH_m), and crystallinity degree (χ_c) of pure PVA and PVA-based systems incorporating 5% (*w/w*), 10% (*w/w*), and 20% (*w/w*) of either pristine or ultrasound-treated lignin.

Sample	T_g (˚C)	T_m (˚C)	ΔH_m (J/g)	χ_c (%)
PVA	106	183	15.9	9.8
PVA/KL05	102	182	13.4	8.7
PVA/KL10	103	182	12.4	8.5
PVA/KL20	111	182	13.3	10.3
PVA/LNP05	103	185	16.0	10.4
PVA/LNP10	105	185	14.3	9.8
PVA/LNP20	115	188	13.6	10.5

All the systems showed a single compositional-dependent T_g, indicating good compatibility between PVA and lignin, thanks to strong mutual hydrogen bonding interactions, as previously observed by ATR–FTIR spectroscopy (Figure S5). Upon incorporation of 5% (*w/w*) and 10% (*w/w*) of either pristine or ultrasound-treated lignin within PVA, little to no variation in the glass transition temperature was recorded for the blends compared to the unfilled polymeric matrix (T_g = 106 °C). On the contrary, slightly higher values of T_g were registered for the formulations at the highest filler loading, up to 111 °C and 115 °C for PVA/KL20 and PVA/LNP20, respectively. When studying the non-isothermal melting behavior of the two-phase systems, T_m was not found to vary in the case of PVA/lignin systems (T_m = 182 °C), regardless of lignin content. On the other hand, as nanolignin loading was increased, the T_m was observed to increase slightly in nanocomposite systems, from 183 °C (neat PVA) to 185 °C and 188 °C for PVA/LNP05, PVA/LNP10 and PVA/LNP20, respectively. This behavior may be associated with hampered free movement and arrangement of PVA backbone chains in the presence of the nanoscale filler[S8]. In addition, the incorporation of pristine lignin into PVA hindered the occurrence of nucleation and/or growth phenomena, as a consequence of poor dispersed-phase distribution within the polymer matrix. As opposed to this, slightly higher values of χ_c were attained in systems incorpoating LNPs, thanks to strong intermolecular interactions and excellent dispersion level achieved in the case of ultrasound-treated nanoparticles, which were able to promote PVA crystallization by acting as nucleation sites[S8]. These trends are in line with recent findings reported in the literature[S8,S12–S14].

Table S5 Values of $T_{5\% \text{ mass-loss}}$, $T_{10\% \text{ mass-loss}}$, $T_{\text{mass-loss peak}}$, and W_{residual} for PVA, PVA/KL, and PVA/LNP films.

Sample	$T_{5\% \text{ mass-loss}}$ (˚C)	$T_{10\% \text{ mass-loss}}$ (˚C)	$T_{\text{mass-loss peak}}$ (˚C)	$R_{750 \text{ °C}}$ (%)
PVA	87	160	333	0.94
PVA/KL05	89	170	340	0.97
PVA/KL10	122	224	338	1.23
PVA/KL20	145	228	336	1.39
PVA/LNP05	140	228	339	1.25
PVA/LNP10	154	232	338	1.29
PVA/LNP20	162	244	337	1.53

References

(S1) https://Support.Minitab.Com/En-Us/Minitab/Help-and-How-to/Statistical-Modeling/Doe/How-to/Factorial/Analyze-Factorial-Design/Methods-and-Formulas/Effects-Plots/.

(S2) Posoknistakul, P.; Tangkrakul, C.; Chaosuanphae, P.; Deepentham, S.; Techasawong, W.; Phonphirunrot, N.; Bairak, S.; Sakdaronnarong, C.; Laosiripojana, N. Fabrication and Characterization of Lignin Particles and Their Ultraviolet Protection Ability in PVA Composite Film. *ACS Omega* **2020**, *5* (33), 20976–20982.

(S3) Korbag, I.; Mohamed Saleh, S. Studies on the Formation of Intermolecular Interactions and Structural Characterization of Polyvinyl Alcohol/Lignin Film. *Int. J. Environ. Stud.* **2016**, *73* (2), 226–235.

(S4) Kubo, S.; Kadla, J. F. The Formation of Strong Intermolecular Interactions in Immiscible Blends of Poly(Vinyl Alcohol) (PVA) and Lignin. *Biomacromolecules* **2003**, *4* (3), 561–567.

(S5) Zhang, J.; Tian, Z.; Ji, X.; Zhang, F. Fabrication Mechanisms of Lignin Nanoparticles and Their Ultraviolet Protection Ability in PVA Composite Film. *Polymers* **2022**, *14* (19), 4196.

(S6) Jipa, I.; Stoica, A.; Stroescu, M.; Dobre, L.-M.; Dobre, T.; Jinga, S.; Tardei, C. Potassium Sorbate Release from Poly(Vinyl Alcohol)-Bacterial Cellulose Films. *Chem. Pap.* **2012**, *66* (2).

(S7) Yang, W.; Ding, H.; Qi, G.; Li, C.; Xu, P.; Zheng, T.; Zhu, X.; Kenny, J. M.; Puglia, D.; Ma, P. Highly Transparent PVA/Nanolignin Composite Films with Excellent UV Shielding, Antibacterial and Antioxidant Performance. *React. Funct. Polym.* **2021**, *162*, 104873.

(S8) Yang, W.; Owczarek, J. S.; Fortunati, E.; Kozanecki, M.; Mazzaglia, A.; Balestra, G. M.; Kenny, J. M.; Torre, L.; Puglia, D. Antioxidant and Antibacterial Lignin Nanoparticles in Polyvinyl Alcohol/Chitosan Films for Active Packaging. *Ind. Crops Prod.* **2016**, *94*, 800–811.

(S9) Kharazmi, A.; Faraji, N.; Mat Hussin, R.; Saion, E.; Yunus, W. M. M.; Behzad, K. Structural, Optical, Opto-Thermal and Thermal Properties of ZnS–PVA Nanofluids Synthesized through a Radiolytic Approach. *Beilstein J. Nanotechnol.* **2015**, *6*, 529–536.

(S10) Coates, J. Interpretation of Infrared Spectra, A Practical Approach. In *Encyclopedia of Analytical Chemistry*; Wiley **2000**, DOI: 10.1002/9780470027318.a5606.

(S11) *Lignin and Lignans as Renewable Raw Materials: Chemistry, Technology and Applications*; Wiley **2015**, DOI: 10.1002/9781118682784.

(S12) Hu, X.-Q.; Ye, D.-Z.; Tang, J.-B.; Zhang, L.-J.; Zhang, X. From Waste to Functional Additives: Thermal Stabilization and Toughening of PVA with Lignin. *RSC Adv.* **2016**, *6* (17), 13797–13802.

(S13) Huang, J.; Guo, Q.; Zhu, R.; Liu, Y.; Xu, F.; Zhang, X. Facile Fabrication of Transparent Lignin Sphere/PVA Nanocomposite Films with Excellent UV-Shielding and High Strength Performance. *Int. J. Biol. Macromol.* **2021**, *189*, 635–640.

(S14) Nair, S. S.; Sharma, S.; Pu, Y.; Sun, Q.; Pan, S.; Zhu, J. Y.; Deng, Y.; Ragauskas, A. J. High Shear Homogenization of Lignin to Nanolignin and Thermal Stability of Nanolignin-Polyvinyl Alcohol Blends. *ChemSusChem* **2014**, *7* (12), 3513–3520.
